# Defining Severe and Persistent Mental Illness—A Pragmatic Utility Concept Analysis

**DOI:** 10.3389/fpsyt.2020.00648

**Published:** 2020-07-06

**Authors:** Naomi Zumstein, Florian Riese

**Affiliations:** ^1^ URPP “Dynamics of Healthy Aging”, University of Zurich, Zurich, Switzerland; ^2^ Department of Anthropology, McGill University, Montréal, QC, Canada; ^3^ Department of Geriatric Psychiatry, Psychiatric University Hospital Zurich, Zurich, Switzerland

**Keywords:** severe and persistent mental illness, SPMI, palliative psychiatry, pragmatic utility concept analysis, systematic review

## Abstract

The concept of severe and persistent mental illness (SPMI) lacks a consensual definition. Variations in definitions stem above all from different meanings about the constituent features of the concept and how to operationalize them. Our objective was to clarify the concept of SPMI and to explore the level of concept maturity through pragmatic utility (PU) concept analysis. Our findings suggest that SPMI is a partially mature concept that needs further clarification. We argue that the lack of a uniform definition is inherent to the problem: SPMI refers to a patient population rather than a disease entity, and the term has to be useful for different stakeholder purposes. Therefore, while an agreement on the principle three dimensions included in a definition may be possible (diagnosis, disability, and duration), their operationalization will have to be context-dependent and specific for the task at hand.

## Introduction

Severe and persistent mental illness (SPMI) is associated with suffering in the affected persons and burden to their caregivers. To provide optimal healthcare for these patients is a challenge. To advance service provision for this population, research is needed to guide treatments and resource allocation. However, several authors have highlighted that SPMI lacks a consensual definition (1–5). Variations in definitions stem above all from different meanings about the constituent features of the concept and how to operationalize them. Consequently, researchers, policymakers, and healthcare providers may have various understandings of SPMI, measure different phenomena, and deal with different patient groups. Therefore, an analysis of the definitional basis of SPMI is needed to advance the field.

The term SPMI was introduced by a workgroup convened by the NIMH in 1987 (6) and defined SPMI as a function of the three “Ds”, namely, diagnosis, disability, and duration. The workgroup aimed to improve service and policy planning in the United States (3, pp. 13–14, 6). In the following years, several authors developed definitions of SPMI to be used on the local level (1–4).

Recently, a debate has emerged that connects SPMI with palliative care (7–15). A paradigm shift to “palliative psychiatry” for SPMI has been proposed as this population is at risk of therapeutic neglect and/or overly aggressive care within the existing care paradigms (13). However, this ongoing debate has so far neglected to scrutinize the closely linked concept of SPMI. SPMI plays a crucial epistemic role in the formation of a palliative psychiatry paradigm as it refers to its target. The current debate about palliative psychiatry marks a “pre-paradigm” stage as the description lacks conceptual consensus (16, p. 47). The idea of palliative psychiatry itself may remain immature as long as the level of conceptual maturity of SPMI has not been assessed. Consequently, there is a need to evaluate the maturity of the concept of SPMI for palliative psychiatry to become “normal science”, i.e., the default state of mature science, which is characterized by a broad consensus of the practitioners of a scientific field on fundamental questions (16, p. 10).

Hence, our objective was to clarify the concept of SPMI in the health sciences and to explore the level of concept maturity through a particular form of systematic literature review, Pragmatic Utility (PU) concept analysis (17–19). Specifically, our research questions were: How is SPMI defined and what are the features of the definition, i.e., is SPMI clearly and consensually defined, fully described, with clear characteristics, demonstrated preconditions and outcomes, and clearly delineated boundaries? The need for a consensus definition of SPMI has emerged in specific domains with particular ways of understanding and reasoning of the issue. Therefore, we also aimed to explore how the problem of defining SPMI was initially framed and followed up by various authors.

## Methods

### Literature Identification and Selection

Following a scoping search, a systematic search of the following databases was carried out to guarantee adequate and efficient identification of pertinent research related to the topic of interest and to minimize publication bias (20): EMBASE, SCOPUS, PsychINFO, MEDLINE, and Web of Science. Search tools such as medical subject headings (MeSH), Boolean operators, truncations, and positional operators were used. The search was limited to English-only literature, and no year limits were applied. The search strategy was developed in consultation with a research librarian, and the search strings adapted to suit the search style of specific databases used. In all databases, single and combined search terms included the terms “severe”, “serious”, “chronic”, “mental”, “illness”, “disorder”, “disease”, as well as “definition”, “concept”, “theory”, “model”, “method”, “concept model”, “concept framework”, and “approach”. The scoping search identified the article by Schinnar et al. (2) as a key article on the topic of definitions of SPMI. Subsequently, a forward search in Google Scholar was performed to identify further studies that may have been missed in the primary database searches for inclusion (21, p. 121).

Only publications that develop, propose, or describe a definition, theory, model, approach, or framework (theoretical or empirical) for defining, assessing, analyzing, and/or reporting SPMI were included for the concept analysis (inclusion criteria). The included studies could be both empirical (qualitative and/or quantitative methods and systematic reviews) or theoretical (theory formation/development based on literature reviews and/or experience). The studies which were identified through the database and citation searches were downloaded into a reference management database and deduplicated. All titles and abstracts were then screened against inclusion criteria. References not meeting the inclusion criteria were excluded. A random sample of 20 articles (10 classified as not relevant, 10 as relevant by the first reviewer, NZ) was drawn, and a blind classification was performed by the second reviewer, FR. Classification differed in only one case that was resolved by subsequent discussion between reviewers 1 and 2, with reviewer 1’s classification prevailing.

### Organization and Structuring of the Literature

Articles were coded using MAXQDA 12 to chart the data. The organization and structuring of the literature were initially undertaken by NZ and then reviewed by FR. Relevant articles were first organized according to general features such as publication year, origin, the field of application, type of studies, and focus of the publication. A more in-depth structuring of the papers was guided by the following analytical questions: Which definitions were used in the literature? Is there a consensus within the literature about the definition? Was the lack of a consensus definition addressed and followed up in the literature? Which definitions of SPMI were used in relationship with the topic of palliative care? Was the lack of a consensus definition addressed and followed up in the literature about palliative care? The coding frame for this in-depth structuring was based on an iterative deductive and inductive category formation (22, p. 93).

### Concept Analysis

We chose a particular variation of a systematic review, namely, PU concept analysis, as developed by Morse et al. (17–19). PU concept analysis explores the level of maturity of concepts by assessing their internal structure, use, representativeness, and/or relations to other concepts. It serves, among other things, to determine the need to refine or clarify a concept or to examine the congruence between the definition of the concept and the way it has been operationalized (17, pp. 75–79). The evaluation of maturity uses the following overlapping criteria: epistemological (clarity of definition, boundaries, attributes, preconditions, and outcomes), pragmatic (operationalization of the concept in research and practice), linguistic (use in various contexts), and logical (differentiation of the concept from other concepts when used in theory). A concept is “mature” when it is well-defined, has clearly described characteristics, delineates boundaries, and documented preconditions and outcomes (18). However, a concept is not required to meet all of these criteria. Between the two extremes of immature and mature concepts are concepts that are partially developed (17, p. 88), for instance, when distinguishing features are not yet fully articulated and need further clarification.

Rather than a series of steps, PU concept analysis is a non-linear, iterative process. It contains, apart from the initial clarification of the purpose of the analysis the following guiding principles (23): a) identification and selection of relevant literature to ensure validity of the concept analysis, b) organization of literature in a general way, c) structuring literature through decontextualization to reveal general features of the concept, and d) formulation of key analytical questions to derive consistent dimensions and boundaries of the concept. Also, we explored how the problem of defining the concept was framed and followed up by analyzing the underlying assumptions and premises.

## Results

### Identified Literature

**Figure 1:**
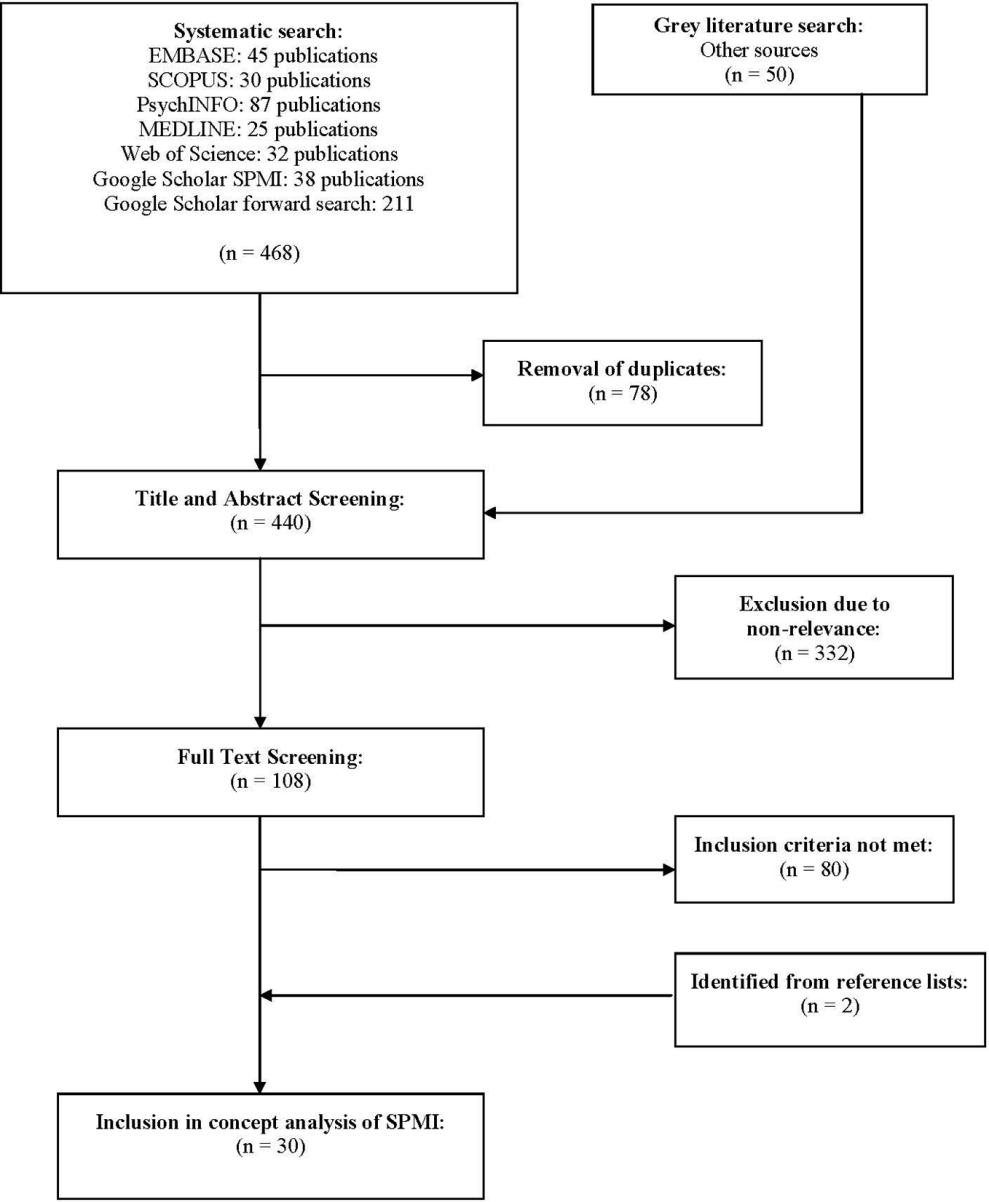
Flow chart of the selection process.

After removal of duplicates, the initial literature search identified 440 articles. After exclusion of articles based on title and abstract, 108 articles underwent full-text reading. Based on the full-text, 28 articles were identified as relevant for our analysis. The reference lists of these studies were checked for further resources, resulting in the inclusion of an additional two publications for a total of 30 articles. For a flow chart of the literature identification and selection process, see **Figure 1**.

The articles in our final sample included descriptive studies (1, 3, 5, 8, 10, 24–32), systematic literature reviews (2, 4, 9, 15), a non-systematic literature review (33), qualitative case studies (11, 34, 35), a mixed-method approach (36), and theoretical papers (7, 12, 13, 37–40). Eleven articles originated in the United States, five in Canada, three each in Australia and Italy, two in Switzerland, one each in Sweden, Taiwan, Belgium, Netherlands, New Zealand, and the United Kingdom. Articles were distributed over time since the initial article in 1990 (2) (see **Table 1**). Definitions and concepts of SPMI in included studies were based on psychiatry (eleven), nursing (six), psychology (three), social science (three), rural, public, population health (three), social work (three), and ethics (one). Findings from our review drawn from the collected articles resulted in the following themes in relation to which SPMI is discussed: health services, care practices, palliative care, recovery, rehabilitation, social support, rehospitalization, and family adaptation. For characteristics of the included articles and the descriptions and definitions used, see **Table 1**.

**Table 1:** Summary table of included articles.

Reference	Publication year	Type of study	Descriptions/definitions of severe and persistent mental illness in the source literature
Schinnar et al. (2)	1990	Empirical	cf. table 1, p. 1604–1605
Schinnar et al. (1)	1991	Empirical	“We select the state definitions of Pennsylvania (PA), New Jersey (N J), New York (NY) because of their geographic contiguity and their use of a SPMI definition to guide the selection of clients for case management programs.”
Veltro et al. (31)	1993	Empirical	“In the present paper the severely and persistently mentally ill, also called ‘continuing care clients’, are defined as ‘people with a two-year history of mental illness or in treatment for two years or more’ (6).”
Durham et al. (26)	1994	Empirical	“This article is concerned with one of the most challenging of those high risk populations: persons with chronic, severe mental illness (SMI). These individuals are defined by the National Institute of Mental Health as having nonorganic psychoses and personality disorders accompanied by major limitations in life activities over a prolonged period of time, thus requiring long term treatment (2, 41).”
Wasylenki et al. (40)	1994	Theoretical	“Severe mental illnesses are defined by diagnosis, disability, and duration (2). Diagnostically they usually include schizophrenic disorders, major affective disorders, and severe personality disorders. In a subpopulation of persons suffering from these disorders, significant disability occurs to the extent that individuals are unable to function in normal social and vocational roles. And finally, a further subpopulation of those who are ill and disabled are chronically afflicted, as measured by duration of symptoms, length of disability, and hospitalization episodes. These three overlapping dimensions—diagnosis, disability, and duration—provide a frame- work by which to delineate the population of severely, persistently mentally ill people in any jurisdiction.”
Rothbard et al. (3)	1996	Empirical	NIMH (6); Schinnar et al. (2); Schinnar et al.(1)
Chandler et al. (25)	1997	Empirical	“The ISAs recruited adults with severe and persistent mental illness into the study group. Study participants had to have a DSM-III-R diagnosis, a functional disability due to the diagnosis, and eligibility for public benefits as a result of the disability.”
Draine et al. (33)	1997	Empirical	“Serious and persistent mental illness is defined as a diagnosis of schizophrenia or a major affective disorder, resulting in lifelong disabling conditions that impair personal and social functioning.” [source of definition unclear]
Slade et al. (4)	1997	Empirical	cf. table 3, p. 179
Hilburger et al. (27)	1999	Empirical	“For this study, a person with severe and persistent mental illness was defined as someone with a DSM-IV Axis I or Axis II diagnosis and who was currently a participant in a psychiatric rehabilitation program.”
Ruggeri et al. (5)	2000	Empirical	NIMH (6); Schinnar et al. (2)
Yamada et al. (32)	2000	Empirical	“Severe and persistent mental illness (SPMI) has been defined in terms of diagnosis, disability, and duration of a mental disorder (42). [… ] The priority population for mental health services consisted of SPMI adults with schizophrenia, major depression, or manic depressive disorder, or other severely disabling mental disorders which require crisis resolution or ongoing and long-term support and treatment.”
Parabiaghi et al. (29)	2006	Empirical	“For the purpose of the present study, we have adopted the two-dimensional definition of SMI proposed by Ruggeri et al. (5): any mental disorder, GAF ≤ 50 and duration of service contact ≥ 2 yrs. A dichotomous classification of psychiatric diagnosis was used, ‘psychotic’ (including the PCR diagnosis of Schizophrenia and functional psychoses and Severe affective disorders) versus ‘non-psychotic’ (including all other PCR diagnoses) cases.”
Pasmeny et al. (30)	2008	Empirical	“Participants met the diagnostic eligibility criteria of SPMI set forth by Parabiaghi et al. (29) and Schinnar et al. (2), which cover diagnosis, duration of illness and severity of disability.”
Woods et al. (15)	2008	Empirical	“While the definition remains open for review, SPMI includes people aged 18 years and older who suffer from a prolonged or recurrent mental illness, are impaired in activities of daily living, and require long-term treatment (5, 43).”
Hsiao et al. (28)	2009	Empirical	“The National Institute of Mental Health (6) defines SPMI using the following criteria: (a) a diagnosis of non-organic psychosis or personality disorder; (b) duration of at least 2 years; and (c) at least three of the following five categories of functional disabilities: dangerous or disturbing behavior, mild impairment in activities of daily living and basic needs, moderate impairment in social functioning, moderate impairment in performance at work, and moderate impairment in non-work activities.”
Arvidsson et al. (24)	2009	Empirical	“The definition of SMI used by the National Board was a person with a mental illness that causes a disability to the degree that it influences daily life. Only persons over 18 years were included. The impairment should have lasted for at least 6 months. Persons with mental retardation and age dementia were excluded (44).”
Koekkoek et al. (35)	2009	Empirical	“We limit the group of non-psychotic chronic patients to those with a severe mental illness (SMI), using the broad definition of Ruggeri et al. (5). This includes all patients that have been in psychiatric care longer than two years and that have a GAF-score at or below 50.”
Torres (38)	2011	Theoretical	“The term serious and persistent mental illness (SPMI) was promulgated by the National Institute of Mental Health (NIMH) during its efforts to formulate a consensus definition (2, 45).”
Terpstra & Terpstra (12)	2012	Theoretical	“Woods, Willison, Kington, and Gavin (15) defined people with SPMI as those 18 and older who experience prolonged or recurrent mental illness, are impaired in activities of daily living, and require long-term treatment.”
Moonen et al. (11)	2016	Empirical	Ruggeri et al. (5), Woods et al. (15)
Trachsel et al. (13)	2016	Theoretical	Ruggeri et al. (5), Woods et al. (15)
Isaacs et al. (34)	2017	Empirical	“Individuals are said to have severe and persistent mental illness (SPMI) when they have ‘severe symptoms or severe difficulty in social, occupational or school functioning’ together with treatment that has lasted for 2 years or more (5).”
Kinter (37)	2017	Theoretical	Goldman et al. (42), Parabiaghi et al. (29)
Banfield et al. (36)	2018	Empirical	Partners in Recovery (PIR) (46)
Butler et al. (8)	2018	Empirical	“SPMI is generally defined in the literature as prolonged or recurrent mental illness experienced by people 18 years and older [e.g., (12)]. Common diagnoses within this population include schizophrenia, depression, bipolar disorder, some personality disorders, post-traumatic stress disorder, and anorexia nervosa (15).”
Elie et al. (10)	2018	Empirical	“SPMI was defined as any DSM-5 mental illness diagnosed for at least 2 years resulting in serious functional impairment (6). Although there are several definitions for SPMI (5), we adapted the definition used by the National Institute of Mental Health, given its inclusive nature.”
Trachsel (39)	2018	Theoretical	“The authors defined SPMI as “any DSM-5 mental illness diagnosed for at least 2 years resulting in serious functional impairment (10)“.
Brown et al. (7)	2019	Empirical	“SPMI refers to adults with prolonged functional impairment from conditions such as schizophrenia, bipolar disorder, depression and some personality disorders (8, 47, 48).”
Donald et al. (9)	2019	Empirical	“Severe persistent mental illnesses (SPMIs) are those that are prolonged and recurrent, impair activities of daily living, and require long-term treatment (15). Common diagnoses include schizophrenia, bipolar disorder, and major depression (15).”

GAF, Global Assessment of Functioning; ISA, Integrated Service Agency; PCR, Psychiatric Case Register; SMI, severe mental illness/serious mental illness; SPMI, severe persistent mental illness.

### Definitions of Severe and Persistent Mental Illness

The analysis of the descriptions and definitions used across the studies reveals an abundance and inconsistent use of different expressions, phrases, and initialisms. For instance, the initialism SMI was not just used for “severe mental illness” but also for “severe and persistent mental illness” instead of SPMI [e.g., (5, 24, 29)]. The initialism SPMI was used for “severe and persistent mental illness” as well as “serious and persistent mental illness” (7). Half of the papers addressed the lack of a consensus definition (1–5, 10, 13, 15, 24, 29–31, 35, 37, 38). Nine articles of those which addressed the lack of a consensus definition also discussed which dimension to include and how to operationalize them (1–5, 24, 29, 31, 38). Initially, the phrase “lack of consensus” referred to a lack of consensus among researchers, mental health planners, and policymakers on the local and national level in the United States (1–3). In subsequent papers, the authors highlighted the lack of consensus on the international level [e.g., (4, 5, 13, 31)]. Hence, the problem of “consensus definition” was reframed in a far-reaching way. Shifting the meaning of the notion “consensus” implies a different approach to find a consensus definition.

Variations in definitions appeared to mainly originate from different meanings of the constituent features of the concept, i.e., which dimensions to include. Associations and forms of the dimensions varied according to the different situations or conditions in which the concept was being used. Commonly, the definitions consisted of three dimensions, namely diagnosis, duration, and disability. “Diagnosis” included a variety of illnesses, “duration” referred either to the duration of symptoms, treatment or disability and “disability” was used for functional impairment or quality of life. Slade et al. (4) proposed a five-dimensional framework for developing definitions at a local level. Ruggeri et al. (5) tested two operationalized definitions in two different settings, a narrow one (the “three-dimensional definition”) based on the National Institute of Mental Health definition (6) and a broad one (the “two-dimensional definition”).

Nine articles discussed SPMI in connection with palliative care (7–13, 15, 39). Four out of the nine papers were theoretical (7, 12, 13, 39) and five empirical (8–11, 15). The definitions of SPMI used in these papers were those from the National Institute of Mental Health (6), Schinnar et al. (2), Ruggeri et al. (5), and Woods et al. (15). Woods et al. refer to Ruggeri et al. (5) and the state definition of serious mental illness of Virginia (USA) (43). Among the palliative care-related articles, only three address the lack of a consensus definition of SPMI (10, 13, 15). However, none of them discussed and followed up on this issue in detail.

### Milestone Publications

We identified the definitions in the papers by the NIMH (6), Schinnar et al. (2), Slade et al. (4), and Ruggeri et al. (5) as the most impactful on subsequent publications and therefore present them in more detail here.

The phrase “severe and persistent mental illness” was introduced by the NIMH in 1987 (6) to replace “chronic mental illness” (CMI) because of the association chronicity has had with continuous or incurable illness (3, p. 10). The task of the workgroup convened by the NIMH in 1987 was to develop a national definition of “severe and persistent mental illness” and builds on the work of Goldman et al. (42), who discussed how to define and count the “chronically mentally ill” (CMI). The objective of the NIMH workgroup was to reduce the variance in counts of persons affected by SPMI to improve service and policy planning and to increase the congruence between federal and state guidelines for beneficiaries of federal and state economic and social support programs (3, pp. 13–14). Also, the workgroup proposed to operationalize the definition using measures of disability or dysfunction that are congruent with the Social Security Administration’s approach to defining disability. However, the workgroup also suggested that specific thresholds on the measures could vary for state and local purposes.

The original NIMH definition (6) comprised the three dimensions diagnosis, disability and duration operationalized as follows: “Diagnosis: A major mental disorder according to DSM-III-R: a major affective, non-organic psychotic disorder or a disorder that may lead to a chronic disability such as a borderline personality disorder. Disability: Severe recurrent disability resulting from mental illness. The disability results in functional limitations in major life activities. Individuals must meet at least two of the following criteria on a continuing or intermittent basis: (1) Is unemployed, is employed in a sheltered setting or supportive work situation, or has markedly limited skills and a poor work history (2) Requires public financial assistance from out-of-hospital maintenance and may be unable to procure such assistance without help (3) Has difficulty in establishing or maintaining a personal social support system (4) Requires help in basic livings skills such as hygiene, food preparation, or money management (5) Exhibits inappropriate social behavior which results in intervention by the mental and/or judicial system; Duration: Treatment history meets one or both of the following criteria: (1) Has undergone psychiatric treatment more intensive than outpatient care more than once in a lifetime (e.g., crisis response services, alternative home care, partial hospitalization, or inpatient hospitalization) (2) Has experienced an episode of continuous, supportive residential care, other than hospitalization, for a period long enough to have significantly disrupted the normal living situation.”

Schinnar and his colleagues (2) used the NIMH definition as a reference point because it reflects a national perspective on SPMI. The authors aimed to define and operationalize “severe and persistent mental illness” to obtain better national and local prevalence rates of SPMI and to assess program effectiveness for recovery from SPMI. Seventeen definitions of the severe and persistent mentally ill, which have been developed in the context of mental health care in the United States were reviewed. Based on the narrative descriptions of SPMI, each criterion was operationalized. The operationalized definitions were applied to a stratified sample of 222 adult patients who were admitted to an inner-city community mental health center in the city of Philadelphia to estimate the prevalence of SPMI in this sample. Their findings suggest that there is general agreement in the literature that diagnosis, disability, and duration criteria in some form are necessary to define serious mental illness. The authors state that there is uncertainty about the relevant diagnostic categories, the nature and degree of disability, the length of illness, and the relative importance of each. According to the authors, there is a general consensus that the duration criterion should reflect the persistence of disability and not the duration of illness or treatment time. The authors posit that departures from the consensus definition, which uses persistence of disability as a criterion for the duration, will and should occur within specific communities with special needs.

In a subsequent study (1), the authors highlight that mental health care needs are too heterogeneous across the United States, and resources available to meet such needs are too unevenly distributed to make a consensus definition of SPMI a practical tool. Hence, federal agencies should promote the development of a consistent framework for counting and reporting services for persons affected by SPMI. The authors underline that states should remain free to develop definitions that suit their local needs best. However, they should be encouraged to develop definitions in a manner that is consistent with the components of the framework.

Slade et al. (4) address the problem of prioritization in mental health care and surveyed current practice in England by obtaining written documentation from 20 agencies on the eligibility criteria they use for deciding whether someone should receive mental health care. The study also included surveys of government departments, user groups, and professional bodies. The authors propose to include a “top-down” and “bottom-up” process in formulating a definition. “Top-down” includes consultation of managers specifying principles for identifying priority groups, by applying official guidelines to the local level. “Bottom-up” entails staff working together to amend these principles in the light of their experience with individual clients. The findings indicate that definitions of severe mental illness are based on the SIDDD dimensions, i.e., safety, informal and formal support, diagnosis, disability, and duration. This particular set of components can be used as a framework for definitions at a local level.

Ruggeri and her colleagues (5) tested two operationalized definitions of “severe mental illness” (SMI) to calculate prevalence rates of SMI in two catchment areas in Europe (South London and South Verona). The prevalence rates were calculated according to a narrow (three-dimensional), and broad (two-dimensional) operationalized definition of SMI, which were derived from the NIMH (6) definition. The “three-dimensional definition” uses three criteria: diagnosis of psychosis, duration of service contact ≥ 2 years, and GAF ≤ 50. The “two-dimensional definition” uses only the last two criteria, i.e., duration and dysfunction. Describing the definition as either “two-dimensional” or “three-dimensional” is, however, imprecise. “Dimension” refers to diagnosis, duration, and disability or, in this case, dysfunction. According to the authors, the two-dimensional definition is based on duration and dysfunction only and applied to patients with and without psychotic disorders. Hence, the dimension “diagnosis” has actually not been excluded but rather operationalized differently.

### Concept Maturity

A concept is “mature” when it is well-defined, has clearly described characteristics, delineates boundaries, and documented preconditions and outcomes (18). The heterogeneity of dimensions included in the definitions did not allow us to assess the preconditions, outcomes, and boundaries of the concept in a structured way. Therefore, we conclude that SPMI is a concept that is only partially mature because definitions and terminology vary widely across the literature and because the constituent features are—as of yet—not fully articulated and need further clarification.

## Discussion

Research and improvement of service provision for SPMI are hampered by a lack of a clear theoretical framework and, consequently, consensus what SPMI actually is. Our aim was, therefore, to systematically review the existing literature on SPMI for its definitions and to perform a PU concept analysis to examine the concept’s maturity. We found inconsistent use of terminology and perpetual confusion about the constituent features of the concept and, consequently, different operationalizations of the dimensions. We conclude that SPMI is—as of yet—a partially mature concept that requires further theoretical development to become useful. Following Brigandt (49), we believe that an essential function of concepts is to set a problem agenda. Also, partial maturity is not a hindrance to practical research. According to MacLeod (50), the open-endedness (or partial maturity) of a concept refers to an as of yet not well-known aspect of reality. However, a concept is not required to give a correct representation of it to be scientifically useful.

### The Lack of a Consensus Definition

Several studies have addressed the problem of how to define and operationalize the concept (1–5, 29, 38). Since the seminal studies of Schinnar and his colleagues (1–3), only Slade at al. (4) as well as Ruggeri and her colleagues suggested how to operationalize the concept (5, 29). Definitions used in articles about palliative care and SPMI refer to four sources (2, 5, 6, 15), and the lack of a consensus definition of SPMI is left commonly unaddressed.

Initially, the phrase “lack of consensus” referred to a lack of consensus among researchers, mental health planners, and policymakers on the local and national level in the United States (1–3). The main goal of finding a consensus definition of SPMI was to increase the congruence between federal and state guidelines for beneficiaries of federal and state economic and social support programs. For instance, the dimension “disability” was formulated in congruence with the Social Security Administration’s approach to defining disability. The Social Security Administration is a national agency of the U.S., and its regulations on disability may not be suitable as a reference point to develop a definition that will be applied in other contexts. Moreover, definitions developed by institutions are commonly not based on empirical findings and not intended for scientific use but for management issues.

Interestingly, these statements about the scale of the definition have been ignored in subsequent studies as the problem of defining “severe and persistent mental illness” (SPMI) or “severe mental illness” (SMI) has been mainly framed as the lack of an international consensus definition. Hence, the problem of consensus definition was reframed in a far-reaching way as it implies a different approach to solving the problem of lack of consensus. The lack of an international consensus definition seems to have become a commonsense idea, a “thought style” (51), which remained unquestioned and carried on from one paper to the other. The underlying assumption of this kind of problematization is that there should be a general theory of SPMI.

We argue that the search for a grand theory of SPMI is misdirected because it ignores that the aim of every individual research project informs the choice of theoretical frameworks and the context-dependence of the operationalization. SPMI is not a disease entity but rather refers to a particular population. Also, the claim for a consensus definition implies a homogeneity of the target population, but all descriptions of the target population are, of necessity, combinations of medical (e.g., diagnostic assessment) and social (e.g., community functioning assessment) (52) as well legal criteria. Hence, the call for a standardized international definition of SPMI is based on an incomplete understanding of the requirements that such a uniform definition would have to fulfill. Moreover, the call for a consensus definition of SPMI follows a kind of reverse epistemology as the elaboration of the concept stands at the beginning of the explanation chain; it precedes the accumulation of empirical evidence.

Finally, while an international consensus definition may facilitate large scale epidemiological research, it may create a clinically irrelevant category on the local level as the definition neglects local conditions, specificities, and nuances and has, therefore, no practical use. SPMI affects people who differ considerably with regards to their diagnoses, treatment histories, functional levels, and needs. Thus, a consensus definition might even be potentially detrimental to the target population, as it may lead to a “one size fits all” approach. This argument has already been put forward by Bachrach (53) but seemingly ignored in subsequent studies. Although she refers to CMI, we believe that her argument also applies to “severe and persistent mental illness”.

### Reaching a Useful Definition

Instead of rebutting the concept of SPMI as a whole due to the alleged problem of lack of consensus definition, we propose to keep it as a heuristic device. Still, we argue against a consensus definition for use on the international level. We argue that the search for a consensus definition of SPMI based on a single general theory is misguided as it ignores the context-dependence of health and healthcare. Future research on SPMI ought to focus first on reducing ambiguity in the terminology and achieving context-dependent clarity about the meaning of the concept (54, p. 10). Hence, to clarify the connection between the concept of SPMI and its theoretical or empirical statements, there is a need to develop a specific context-dependent theoretical framework and to formulate a definition of SPMI for use on the local level and to operationalize the constituent dimensions according to specificities and needs in a given context.

As a starting point for such locally useful definitions, we propose the original NIMH definition of SPMI (see results section) (6). According to van der Steen (54, p. 18), a useful definition in the life sciences must be clear, not circular, neither too broad nor too narrow, should not include accompanying features, and should refer to the features present (rather than referring to features that are absent). Accordingly, we suggest the definition of SPMI to be based on the three Ds, i.e., diagnosis, duration, and disability. As constituent characteristics, they are abstract enough to define the concept regardless of the context in which it appears, yet unique enough to define and differentiate it from other concepts [cf. (18, p. 388)]. Also, this allows a notion of SPMI that is wide enough for quite different further developments. Given the context-dependence of the different constituent features of the definition, we argue that the constituent dimensions then have to be further operationalized according to the priorities, needs, and subtleties prevailing in a given context. Local operationalizations would undoubtedly require an update of the diagnostic system in use (i.e., DSM 5 or ICD-11 instead of DSM-III-R) and would likely involve more subjective parameters in the disability and duration dimensions (emphasizing SPMI as illness concept rather than as healthcare planning construct).

### SPMI and Palliative Psychiatry

It has been postulated that a palliative approach in psychiatry has the potential to improve the quality of care, person-centeredness, and autonomy for patients affected by SPMI (13). In other words, SPMI is considered the target population of palliative psychiatry, and palliative psychiatry attempts to promote person-centeredness and patient autonomy. As to be expected from our concept analysis, a recent article on a novel therapeutic approach to SPMI had to further specify its intended target population as “most serious forms of SPMI, for instance, when the quality of life is seriously compromised” (55). We ultimately believe that a definition of SPMI true to a palliative ethos of care will necessarily have to take the perspectives of the affected persons into account. Thus, a definition of SPMI for use in the field of palliative psychiatry should be negotiated among user groups and their caregivers, government agencies, insurers, and treatment teams who establish a set of principles to help guide the development of a definition. To a certain extent, this may allow for the self-assignment of persons affected by SPMI to receive palliative care in psychiatry based on patients’ needs, goals, and preferences. However, the theoretical framework, as well as the empirical evidence for palliative psychiatry, have to be developed further to be able to define its target population.

Hence, we propose a participatory/collaborative approach as a research strategy which combines theoretical and practical knowledge to generate new knowledge about a phenomenon. The underlying ethos of such a research strategy has been formulated by Burns (56). The work of Vreman et al. (57) illustrates how semi-structured group discussions and open session workshops among various stakeholders provided insights about the concept of unmet medical need (UMN).

## Strengths and Limitations

This is the first PU concept analysis to evaluate the maturity of the concept of SPMI. Important strengths of our systematic review include a well-defined research question, an exhaustive search strategy, well-defined inclusion and exclusion criteria, and comprehensive data coding. A significant limitation was that the iterative coding process was mainly performed by NZ. However, the analysis of the coded data was conducted in close cooperation with both authors.

## Conclusions

A systematic concept evaluation is a fundamental step in the research process that should precede more formal research procedures such as operationalization or identification of the variables (18). It allowed us to identify ambiguities and inconsistency of constituent features of the concept of SPMI. According to our PU concept analysis, SPMI refers to a partially mature concept that lacks a standard definition. We argue that this lack of a uniform definition is inherent to the problem: SPMI refers to a patient population rather than a disease entity, and the term has to be useful for different stakeholder purposes. Hence, the next step toward concept clarification and development consists of an assessment of the different perceptions of various stakeholders through a participatory oriented approach between researchers, persons affected by SPMI, and multiple practitioners. Insights gained from semi-structured group discussions and open session workshops among these different stakeholders have then to be incorporated in a modified definition of SPMI.

## Data Availability Statement

All datasets generated for this study are included in the article/supplementary material.

## Author Contributions

All authors contributed to the article and approved the submitted version.

## Funding

Open access publication was funded by the University of Zurich.

## Conflict of Interest

The authors declare that the research was conducted in the absence of any commercial or financial relationships that could be construed as a potential conflict of interest.
